# Emerging Non-Thermal Technologies as Alternative to SO_2_ for the Production of Wine

**DOI:** 10.3390/foods10092175

**Published:** 2021-09-14

**Authors:** Filipa V. M. Silva, Sanelle van Wyk

**Affiliations:** 1 LEAF, Instituto Superior de Agronomia, Universidade de Lisboa, Tapada da Ajuda, 1349-017 Lisboa, Portugal; 2 Department of Chemical & Materials Engineering, University of Auckland, Private Bag 92019, Auckland 1142, New Zealand; sanellevanwyk@yahoo.com

**Keywords:** sulfur dioxide, pasteurization, pulsed electric field, high pressure processing, microbial inactivation, *Brettanomyces*

## Abstract

SO_2_ is an antioxidant and selective antimicrobial additive, inhibiting the growth of molds in the must during the early stages of wine production, as well as undesirable bacteria and yeasts during fermentation, thus avoiding microbial spoilage during wine production and storage. The addition of SO_2_ is regulated to a maximum of 150–350 ppm, as this chemical preservative can cause adverse effects in consumers such as allergic reactions. Therefore, the wine industry is interested in finding alternative strategies to reduce SO_2_ levels, while maintaining wine quality. The use of non-thermal or cold pasteurization technologies for wine preservation was reviewed. The effect of pulsed electric fields (PEF), high pressure processing (HPP), power ultrasound (US), ultraviolet irradiation (UV), high pressure homogenization (HPH), filtration and low electric current (LEC) on wine quality and microbial inactivation was explored and the technologies were compared. PEF and HPP proved to be effective wine pasteurization technologies as they inactivate key wine spoilage yeasts, including *Brettanomyces*, and bacteria in short periods of time, while retaining the characteristic flavor and aroma of the wine produced. PEF is a promising technology for the beverage industry as it is a continuous process, requiring only microseconds of processing time for the inactivation of undesirable microbes in wines, with commercial scale, higher throughput production potential.

## 1. Introduction

Oxidation and the undesirable activity of specific microorganisms have a negative effect on wine quality and shelf life. Sensory quality is the most important factor for wine consumers. Most sensory related attributes are largely dependent on wine’s phenolic composition which determines color, bitterness and astringency [1]. The loss of wine quality during storage is often accelerated due to exposure to sunlight, high temperatures, oxygen, vibration, pH, contaminants from the storage environment surrounding the wine or cork, microbial spoilage and the failure of bottle closures. The storage of wine for ageing and maturation depends on chemical composition and equilibria, with specific flavors and characteristics, which increase wine quality, developing during this period. On the contrary, the quality of white wines typically does not improve during storage, so they can be sold and consumed straight after production within the first 1 to 2 years. On average, red wines have a longer shelf life than white wines due to their higher phenolic concentration, which reduces their susceptibility to oxidation.

In winemaking, sulfur dioxide (SO_2_) is often used at different stages in the production process (e.g., after harvesting the fruit, after crushing, added to the must before fermentation, before maturation, before bottling). SO_2_ has the ability to control oxidative processes including polyphenol oxidase and to inhibit Maillard reactions. If left untreated, oxidation can lead to a decrease in the sensorial and nutritional quality of wine [2,3]. In addition to antioxidant action, SO_2_ also exhibits antimicrobial capacity against spoilage microorganisms, inhibiting the growth of molds in the must during the early stages of wine production, as well as undesirable bacteria and yeasts during fermentation, preventing unwanted secondary fermentation and the formation of yeast haze [4], and thus avoiding microbial spoilage during wine production and storage. The addition of SO_2_ to wine before bottling leads to an increased shelf life, with less likelihood of the formation of off-odors. SO_2_ exists in a bound and free form, the latter being the active form of the compound. The amount of each form present depends on the pH of the wine [3,5]. As wine pH increases, antimicrobial capacity decreases. The addition of SO_2_ can also increase the extraction of phenolic [2,3].

The excessive use of SO_2_ can have a detrimental effect on wine quality including the neutralization of wine aroma, the formation of hydrogen sulfite, unwanted aromas and flavors and cloudiness after bottling [3,6]. Moreover, SO_2_ can have adverse effects in humans including allergic reactions, headaches, asthma, dermatitis, abdominal pain, diarrhea and bronchoconstriction. As SO_2_ is a commonly used preservative in the wine industry, it is also important to consider the cumulative effect it has on the consumer [4]. This led to the establishment of strict regulations and limits governing SO_2_ application in wineries. The SO_2_ regulatory limits for wine preservation are constantly being reviewed and reduced [2,3]. Currently, the International Organization of the Vine and Wine (OIV) recommends 150 mg/L total SO_2_ for red wines, the European Union limits the total use of SO_2_ to 160 mg/L for red wines and 210 mg/L for white and rosé wines and Australia permits 350 mg/L total SO_2_ for all wines [3]. The use of fungal-source chitosan for the inactivation of *Brettanomyces* has also been authorized by the OIV and European Union (Regulation (EC) No 606/2009) [7]. Although SO_2_ free wines are considered healthier, more natural and sustainable, it is a challenge to produce wines without the addition of SO_2_. Consequently, the wine industry is interested in finding alternative strategies to reduce or eliminate SO_2_ in wine production, while maintaining wine quality. To be successful, the alternative must provide the same level of microbial stability and antioxidant activity while also safeguarding the quality of the wine produced, and be less harmful to humans [3]. The use of thermal technologies is unacceptable for the wine industry because of their detrimental effects on the delicate organoleptic characteristics of wine (e.g., flavor, aroma and color) [8]. Thus, the application of non-thermal technologies to produce, age and preserve wine is an area of great interest. Ideally, these technologies will allow the reduction in the use of SO_2_ additive in wine production, while keeping or improving the original characteristics of the produced wine [4,9,10,11,12]. Van Wyk et al. (2018) compared sensory, microbiology and other quality parameters in wine subjected to SO_2_ addition, HPP and PEF treatments during one year storage [13]. No sensory differences were detected between HPP and PEF treated wines and the untreated wines after being stored for one year [13]. The inactivation of polyphenoloxidase enzyme by US, PEF and HPP has been demonstrated [14], and this could be another way to control the undesirable change in the polyphenol profile of wines.

In this investigation, a review of the application of the following non-thermal technologies for wine pasteurization was carried out: pulsed electric fields (PEF), high pressure processing (HPP), ultrasound (US), high pressure homogenization (HPH), low electric current (LEC), ultraviolet irradiation and filtration. The specific objectives were: (i) to present the fundaments of the non-thermal technologies mentioned and their benefits in terms of wine quality; (ii) to review and introduce the main microorganisms of concern that can potentially spoil wine; (iii) to investigate the effect of non-thermal technologies on microbial inactivation and compare the technologies in terms of the efficiency of key microbes’ inactivation in wine; (iv) to discuss the commercial viability of using non-thermal technologies to reduce or eliminate the use of sulfur dioxide in the wine industry.

## 2. Non-Thermal Cold Pasteurization Technologies for Wine Preservation

### 2.1. Pulsed Electric Fields (PEF)

**Figure 1:**
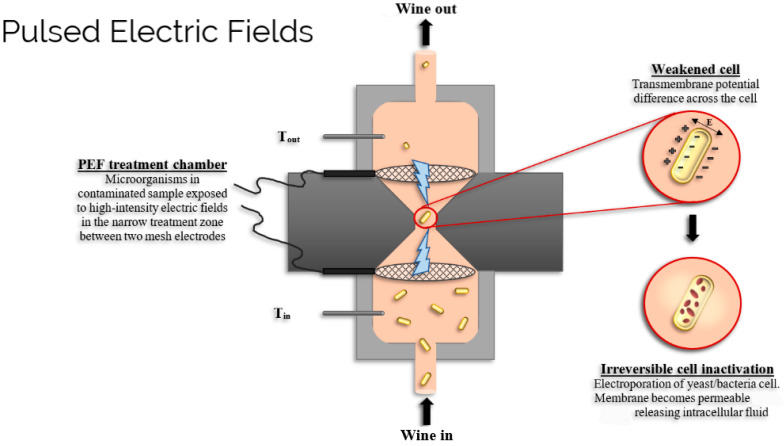
Diagram showing the pulsed electric field (PEF) inactivation of wine spoilage microorganisms (T refers to temperature which is below 40 °C for a non-thermal process).

PEF, a relatively novel non-thermal technology, is a promising alternative for wine preservation because it operates in continuous mode [4] (Figure 1). Depending on the application, it can have a relatively low energy consumption compared to other food preservation technologies [15]. Since PEF is a continuous process, it is easier to integrate into existing industrial processes. In operation, short microsecond pulses of high electric field strength are applied to pumpable beverages flowing between two electrodes [16]. The shape of the applied pulses is either exponential, where the voltage rises quickly to its maximum before decaying slowly to zero, or square where it remains constant for a few microseconds [16,17,18].

A typical PEF system is based on a high voltage pulse generator along with a treatment chamber, a suitable fluid handling system, and monitoring and controlling devices [4,16]. PEF is able to inactivate spoilage microorganisms, often without any significant effect on beverage quality [16,17]. Figure 1 shows an example of a PEF unit including details of some components (e.g., PEF treatment chamber, electrodes, thermocouples) and also information on the mechanism of microbe inactivation in the beverage. Microbial inactivation is thought to occur due to the formation of a potential difference across the microbial cell membranes. This in turn causes the permeabilization or electroporation of the cell membrane, leading to the loss of intracellular fluids [19]. The electric field strength required for the inactivation depends on the size and shape of the cell as well as the composition of its membrane. The bigger the size of the cells, the more susceptible the cells are to the applied electric field pulses; for example, vegetative yeasts are more susceptible than vegetative bacteria cells [20]. Rod-shaped cells required five times stronger electric fields than spherical cells of similar dimensions [19].

Most authors have reported a temperature increase associated with increasing treatment electric field strength or energy [16]. This increase in temperature also increases the rate of inactivation by improving the cell membrane fluidity and mass transfer from the cells [19]. As this temperature increase is not desirable for wine, proper processing conditions must be selected to ensure a non-thermal process, namely flow rate, initial wine temperature and electric field intensity.

Examples of PEF processed beverages include grape-, strawberry-, orange- and apple juices, milk, soup, liquid egg and beer [15,16,18,20,21,22]. PEF can also be operated or designed as a batch process, thus cured fish and meat products have also been processed [23]. One of the main reasons why PEF has not been commercially implemented is the need for high-voltage electric pulses, which pose a significant safety risk in commercial scale operations.

#### Impact on Wine Quality

Abca and Evrendilek (2014) [20] found that subjecting red wine to three µs bipolar PEF pulses at 31 kV/cm (T ≤ 40 °C), resulted in no changes to sensory quality and the treatment had no effect on total phenolic content, antioxidant activity or color. Using 100 pulses of the same electric field strength (31 kV/cm), Puértolas et al. (2009) [17] concluded that the color and odor of PEF treated red wine remained unchanged. Red wine processed using 20 kV/cm (T ≤ 42 °C) for 10,000 µs, had no effect on the anthocyanins present in the wine, but led to a slight increase in tannin concentration and slight changes in the total phenolic index and color intensity [19].

PEF also has other applications in wine production. The technology has been applied to grape pomace and grape skins during the early stages of wine production to improve final wine quality or reduce maceration time. Puértolas et al. (2010a) [24] concluded that a pilot-scale PEF unit was able to produce a high phenolic content wine that can subsequently be used to create a high-quality oak aged red wine. The crushed Cabernet Sauvignon grapes were treated using 50 pulses at 5 kV/cm (122 Hz, 3.67 kJ/kg, <30 °C), after which potassium metabisulfite was added before fermentation [24]. The treatment of Cabernet Sauvignon grape juice with skins using square pulses at 5 kV/cm for a residence time of 0.41 s (122 Hz, 50 pulses, 3.67 kJ/kg, ≤ 17 °C), produced a finished wine that required a shorter maceration time and had a higher Folin-Ciocalteu index, polyphenol concentration and color intensity [22]. López et al. (2008) [25] tested the effect of PEF treatment (10 kV/cm, 50 pulses, 1 Hz, 6.7 kJ/kg, <30 °C) of grape skins on wine quality factors. The prior PEF treatment reduced the duration of maceration during vinification and increased the quantity of phenolic compounds in the final wine. A low electric field strength of 5 kV/cm to treat Cabernet Sauvignon grape pomace led to an increase in the color intensity, total phenolic content, tannins and anthocyanins of the final wine produced [26]. López et al. (2009) also found that the PEF treatment of grape pomace was able to reduce the required maceration time from 268 to 72 h [26].

### 2.2. High Pressure Processing (HPP)

**Figure 2:**
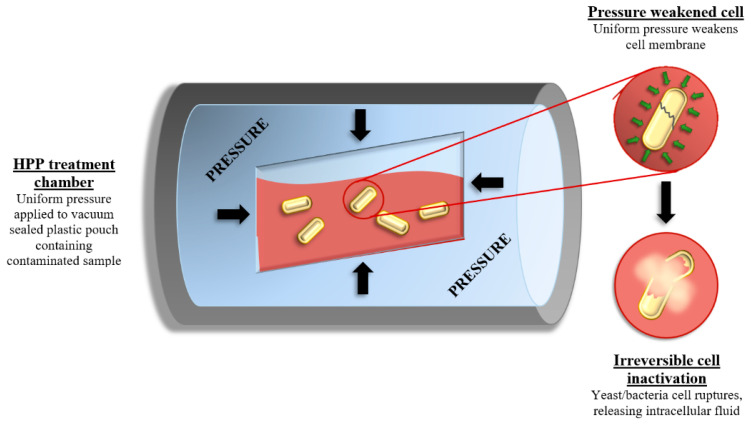
Diagram showing the high-pressure processing (HPP) inactivation of wine spoilage microorganisms.

HPP is a modern commercial non-thermal technology that can be used to extend shelf life by inactivating spoilage and pathogenic yeasts, molds and bacteria in wine, beer, juices, solid foods and other products through the application of isostatic and uniform pressures usually between 100 and 600 MPa [4,27,28,29,30,31,32,33,34,35]. Microbial inactivation can generally be increased by increasing pressure or holding time [29,30,35]. According to previous research, HPP has a greater effect on larger molecules such as enzymes, proteins, and lipids, whereas smaller molecules including vitamins, flavor and several bioactive/health related compounds are generally unaffected. Thus, HPP can better maintain the quality of food, without any significant impact on flavor [32,36,37,38]. Microbial inactivation can occur due to the disruption of cell membranes, protein denaturation and solute loss (Figure 2). Research suggests that microbial inactivation by HPP involves the perforation of the cell membrane, the formation of dimples and swellings or the overall shrinkage of the volume of the cells [35,39,40]. The financial feasibility of HPP is also greatly increased by using the shortest possible processing time that still achieves the desired level of pasteurization [4].

A HPP unit consists of a vessel, a pressure generating pump and a yoke for safety. In the HPP chamber, pressure is applied uniformly throughout a pressure medium and the sealed product. Usually, vacuum-sealed flexible pouches are used for the “in pack” process, as they can withstand both a decrease in volume during compression and the associated expansion on decompression. More recently, Hiperbaric developed a technology named “in bulk” HPP for processing liquids before packaging, allowing the use of any type of rigid container (e.g., glass, can, etc.). Due to the adiabatic compression inside the chamber, a temperature increase of around 3 °C per 100 MPa occurs [11,28]. The increase in temperature is dependent on the properties of the food and can be reduced by decreasing the rate of pressurization [4]. The maximum temperature after compression can be reduced by starting the process with a low initial temperature of the beverage.

Most HPP units are operated in batch, but semi-continuous units for pumpable products have now also been developed by industry [41]. Due to HPP being independent of the shape and size of the product, the scale-up to commercial size is relatively simple. When HPP is unable to produce the required level of microbial inactivation, it can be combined with reduced quantities of preservatives or other preservation technologies [42].

Since 2000, the usage of HPP technology worldwide has increased exponentially and has been implemented commercially in Japan, the USA and across Europe. At the end of 2010, the annual world-wide HPP production capacity reached 250,000 tons. The total number of installed HPP units has been increasing quickly in the food and beverage industry: 315 in 2015, >400 by 2016, etc. [41]. The current industry suppliers of HPP units include Avure (Ohio, USA), Hiperbaric, S.A. (Burgos, Spain), Uhde High Pressure Technologies GmbH (Hagen, Germany) and BaoTou Kefa High Pressure Technologies (BaoTou, China). In 2013, Hiperbaric and Avure introduced the largest HPP unit with a capacity of 525 L and throughput of 3000 L or kg per hour [41]. HPP processed products currently available on the market include fruit and vegetable juices/smoothies, other beverages, pre-cooked meals and meat- and fish-based products [41,43,44]. The application of HPP is currently limited to high-value products due to the high initial capital investment required [41]. The cost of production must therefore be offset by the increase in the value of the product produced through an increase in quality, shelf life, consumer convenience, reduction in preservatives and lower transportation, labor or storage costs. In conclusion, the advantages of HPP include: (i) operation at or below room temperature; (ii) microbial inactivation with no heat damage and fewer preservatives and other additives, improving sensorial and nutritional quality; (iii) instantaneous transfer of pressure, regardless of liquid size and geometry; and (iv) the creation of novel functional food properties, including new structures and textures [45]. This makes HPP an attractive alternative technology for use in the wine sector.

#### Impact on Wine Quality

In 1996 Lonvaud-Funel et al. [46] investigated the effect of high pressure on microbial inactivation in wine for its stabilization. HPP treatment of 650 MPa for 15 min caused no change in the overall sensory quality of red wine and 11 sensory attributes [47]. The same study found that total phenolic content, tartaric esters, flavonols and tannins decreased when wine was subjected to the same HPP treatment. Using milder conditions of 500 MPa for 5 min, Santos et al. (2013a) [48] found no change in the antioxidant activity and b* color parameter of red wine directly after processing. Storing the wine in bottles caused minor changes in the wine sensory properties after 9 months, and higher L*, a* and b* color parameters and lower antioxidant activity after 12 months in-bottle storage [48]. Puig et al. (2003) [9] detected no effect on organoleptic quality when subjecting red wine to the same conditions (500 MPa, 5 min). Mok et al. (2006) [27] found that HPP treatment at 350 MPa for 10 min had no effect on the mouthfeel, aroma and taste of red wine.

Regarding white wine, Puig et al. (2003) [9] detected no organoleptic difference between untreated and 500 MPa, 5 min treated wine. Santos et al. (2013b) [49] found that the same HPP conditions had no effect on the antioxidant activity and total phenolic content of white wine. Processing liquor white Sauterne wine using 355 MPa for 10 min resulted in no significant difference in the sensory quality [50]. Red grape must subjected to 551 MPa for 10 min did not have any effect on the color and little or no effect on the sensory quality of the final wine produced [51].

Research has shown that HPP can lead to the acceleration of wine ageing [45,48]. This suggests that HPP has the potential to shorten the time required to produce a desirable, high quality ‘aged’ wine. HPP can also change wine color involving decreased color intensity and an increase in brownish color often associated with aged wines [48,49].

### 2.3. Power Ultrasound (US)

Power ultrasound (US) is a non-thermal technology with the potential to be used as a wine preservation technique. Sound intensity or acoustic intensity between 10 and 1000 W/cm^2^ [52,53,54] producing waves with frequencies between 20 and 100 kHz are applied to liquid samples causing the formation of small bubbles. When these bubbles collapse, a phenomenon known as cavitation occurs. Localized high pressure (50,000 kPa) and temperature (5000 °C) regions are formed as well as free radicals, shock waves and shear forces. High frequency small bubbles result in a less intense but more uniform acoustic field with a greater rate of cavitation collapse during sonication [55]. In ultrasound units, a generator converts electricity into high-frequency alternating current, followed by the conversion of current into mechanical vibrations [56]. Liquid foods are sonicated by the direct contact of a probe, also known as a sonotrode, horn, finger or ultrasonic tip [56]. US units have also been designed to operate in batch or continuous mode, with cooling water jackets to control temperature during operation [57], as a temperature increase is expected [55,58,59]. The microorganisms are inactivated due to the disruption of their cell membranes/walls. The technology has also been found to be good for food deteriorative enzyme inactivation (e.g., polyphenoloxidase) [14,56,58]. Cavitation may affect the chemical and physical properties of processed foods and can accelerate the wine aging process [15,60].

The efficiency of US is dependent on the viscosity, vapor pressure and surface tension of the liquid sample treated. Microbial inactivation by US also depends on the shape, size and species of microorganism treated, with smaller cells believed to be less sensitive than larger cells [53,55,61,62].

Potential uses of US in the wine industry include wine aging/maturation, improved fermentation, sanitation of barrels and equipment, and the extraction of bioactive aromatic and phenolic compounds from grapes and must. US can also be used to improve the penetration of the wine into the structure of oak barrels and chips, improving oak vanilla, caramel, cream, earthy and spice flavors [4]. US has been found to be more effective at sterilizing wine barrels compared to existing methods, while also providing a significant cost reduction [55]. As this technology is already used commercially for food applications (e.g., extraction of compounds, emulsification, and degassing), it should be easy to scale up for pasteurization purposes. According to Gracin et al. (2016) [54], the scale-up of continuous flow US is possible, which significantly improves its ability to be utilized in the wine or other beverage industry.

#### Impact on Wine Quality

Singleton and Draper (1963) [63] found that using ultrasound treatment at 90 kHz for 60 min in direct contact with wine, increased the tannin concentration and changed the overall flavor of red wine. Indirect water bath ultrasound treatment (30 kHz, 20 kPa) of red wine over a prolonged period of 10 days resulted in increased anthocyanin concentration, and a decrease in L* color parameter [64]. Similar to Singleton and Draper (1963) [63], Luo et al. (2012) [53] found that treating red wine (24 kHz, 0.2 W/mL, 22 mm radius probe) led to significant changes in flavor and aroma. Zhang and Wang (2017) [65] investigated the effect of US on red wine directly after treatment and also after 70 days storage. Directly after US processing using 20 kHz, 950 W for 14 min, color density and visual characteristics increased. After storage, the trend in changing phenolic composition was similar in the US treated and untreated wine. Lastly, Singleton and Draper (1963) [6] studied the effect of US on white wine. Treatment conditions of 90 kHz and 35 W for 60 min resulted in an increase in tannin concentration as well as the generation of a ‘scorched’ off-flavor. One other potential application of US in the wine industry is the acceleration of wine maturation. Ultrasound treatment was found to accelerate changes in wine quality associated with wine aging, thereby shortening vinification time [63,65].

### 2.4. Ultraviolet (UV) Irradiation 

UV light is an energy efficient technology that employs the use of UV-A (320–400 nm), UV-B (280–320 nm) and UV-C (200–280 nm) radiation for microbial inactivation [5]. The UV microbial inactivation mechanism is thought to involve damage to microbial cell DNA, inhibiting reproductive and other cell functions [5]. UV-C penetrability and effectiveness depend on the absorbance, color, density, soluble solids and suspended materials in the beverages [5,66]. Industrial applications of UV include the disinfection of water, surfaces and food packages.

#### Impact on Wine Quality

Falguera et al. (2013) [6] used UV-visible irradiation (250–740 nm, 0–210 min, 25 °C) to process 800 mL of white wine must. The same level of preservation (polyphenoloxidase inactivation) was achieved as with the use of SO_2_ without a significant change in quality parameters including pH, alcohol and tartaric acid content. The ability of UV-C (254 nm) to inactivate yeasts (*Brettanomyces bruxellensis* and *Saccharomyces cerevisiae*) and bacteria (*Lactobacillus plantarum, Pediococcus acidilactici, Oenococcus oeni* and *Acetobacter aceti*) in grape juice and wines was investigated [66]. Dosages of up to 3672 J/L (flow rate of 4000 L/h) were able to cause microbial log reductions within a range of 4.3 to 5.0 in Chardonnay and Pinotage wines, and Chenin Blanc and Shiraz juices. UV-C was able to stabilize the grape juice and wine, reducing the required level of SO_2_ [66]. However, wine color deteriorated as polyphenol-oxidase activity was not prevented, so a residual use of SO_2_ was still necessary [6]. As expected, ultraviolet was more effective in white wine than red wine [5].

### 2.5. High Pressure Homogenization (HPH)

HPH is mainly known for preventing milk creaming in homogenized milk. The main differences between HPP and HPH is that the latter can be used in a continuous operation mode and employs much lower pressures than HPP. 

#### Impact on Wine Quality

Comuzzo et al. (2015) [67], found that HPH treatments between 50 to 150 MPa produced wine with stronger fruity aromas and less off-flavors. Additionally, multiple HPH passes at 200 MPa resulted in no significant impact on the sensory attributes of red and white grape musts. HPH technology can also be used to disrupt and break microbial cell membranes [68].

### 2.6. Filtration

Filtration has the ability to decrease microbial growth, reduce browning and prevent haziness through the removal of colloids [69]. The suitable pore size for wine filtration depends on a number of factors including the effectiveness of removal of targeted microorganism, throughput and economic viability. To remove yeasts and bacteria, it is generally recommended to use a pore size of 0.45 µm [69]. However, it has been reported that the cell size of yeasts such as *B. bruxellensis* decreases as a result of SO_2_ treatment [70]. As a result, filtration using 0.45 µm pore size after SO_2_ treatment could be insufficient for the removal of damaging spoilage microorganisms such as *B. bruxellensis* [71]. Due to membrane fouling, wine quality and permeation rates are often reduced. This in turn decreases the economic viability of filtration [19,69]. 

#### Impact on Wine Quality

Filtration can often have a detrimental effect on wine characteristics, most significantly sensorial properties [43,69]. The smaller the filter/membrane pore size, the greater the of negative impact on wine quality. Due to its effect on the colloidal structures of wine, filtration reduces the color intensity, viscosity and body of wine [17,72,73]. Arriagada-Carrazana et al. (2005) [74] conducted a study investigating the effect of membrane filtration on the phenolic quality and aromatic profile of a Cabernet Sauvignon wine. A 1.2 µm pre-filter followed by a 0.65 µm filter led to a reduction in color intensity and polyphenolic profile. A sensory panel, conducting a triangle test, confirmed the reduction in color and polyphenolic quality. Membrane adsorption was thought to have caused the observed difference in wine quality [74].

### 2.7. Low Electric Current (LEC)

As opposed to PEF, LEC uses a constant low-power electric charge (≤ 200 mA) over prolonged treatment times up to several months to reduce the integrity of the membranes of spoilage microorganisms [69]. 

#### Impact on Wine Quality

LEC treatment of red wine using 200 mA for 60 days had no effect on wine color or odor [73]. The ability of LEC (200 mA, 90 days, <15 °C) to prevent the formation of undesirable flavors by *D. bruxellensis* during storage in oak barrels was compared to SO_2_ treatment [75]. A sensory panel of 18 analyzed the wines after four months in the barrels and found that there was no difference between the two wines in terms of phenolic content or sensory properties. Low-voltage treatment was shown to have a positive effect on grape juice fermentation by subjecting SO_2_-free grape must to 200 mA for 16 days [76]. LEC had no effect on the growth of *S. cerevisiae* while increasing the death rate of apiculate (lemon-shaped cell morphology) yeasts.

## 3. Microbial Wine Spoilage

The microbes are the main targets of the preservation technologies presented in the following Section (Section 4—Effect of PEF, HPP and other non-thermal technologies on microbial inactivation in wine). This section is based on a previous publication by Van Wyk and Silva (2019) [4]. Yeasts and bacteria are common types of wine spoilage microorganisms which can have negative effects on wine quality and shelf life, leading to detrimental economic losses. As the yeast *Saccharomyces cerevisiae* is generally more tolerant to high ethanol concentrations compared to other microorganisms [77], it is widely employed for several industrial fermentation processes, including the production of alcoholic beverages. With respect to wine, *S. cerevisiae* is the most abundant microorganism found in the final wine at the end of fermentation, converting the must sugars into alcohol and generating important compounds (e.g., aroma), which are vital for the final wine properties. However, it is important to control the activity of this oenological yeast after fermentation to keep the wine desirable properties and stability during storage. This fermenting yeast can be controlled in wine by SO_2_ additions or inactivation with non-thermal processes, as investigated by a number of authors.

Contaminant yeasts detected in wines belong to the genus *Brettanomyces/Dekkera, Saccharomyces*, *Schizosaccharomyces* and *Zygosaccharomyces*, while spoilage bacteria include *Lactobacillus*, *Leuconostoc*, *Oenococcus*, *Pediococcus* and *Acetobacter* [2]. Off-odors, haziness and precipitation are indicators of microbial spoilage. The source of contamination can be the grape skins, insects, winery walls and other equipment in contact with the grapes, grape juice, must or wine. As red wines’ grape skins and stems are in contact for longer periods of time with the juice/wine during fermentation, these wines are more frequently contaminated than any other wine type [2,78,79]. The wine industry prevents microbial contamination by ensuring grape quality, proper sanitation of winery equipment including oak barrels, and controlling oxygen and sulfite levels [79].

### 3.1. Brettanomyces Yeast

*Brettanomyces* or *Dekkera* (name given to the spore forming microorganism) poses a great threat to the wine industry, leading to detrimental economic losses worldwide [2,79,80]. This yeast has been detected in wines and wineries across the world, including all major wine producing countries [79,80]. The genera *Brettanomyces/Dekkera* consists of five species: *B. custersianus*, *B. naardenensis*, *B. nanus*, *B. anomalus* and *B. bruxellensis* [80]. *B. bruxellensis* is mainly found in barrel-aged red wines with low SO_2_ content and high pH. The presence of less than 10^4^ cfu/mL can have a detrimental effect on the sensory quality of wine, resulting in unpalatable off-odors and -flavors [2,78,79]. *Brettanomyces* yeasts are infamous for causing mousy off-flavors, also known as ‘*Brett*. character’. The off-odors produced are characterized as being ‘barnyard-like’, ‘medicinal’, ‘Band-aid^®^’ or ‘horsey’. The chemical compounds responsible for the off-flavors and -odors are 4-ethylguaiacol (4-EG) and 4-ethylphenol (4-EP). *Brettanomyces* is the only known microorganism to cause the formation of these compounds in wines [78,79,80,81]. Depending on the species and strain, *Brettanomyces* can also cause the formation of acetic acid known as vinegar taint. When volatile fatty acids are produced, they can cause rancid and/or cheesy flavors and odors [80,81]. Survival studies conducted by Barata et al. (2008) [82] found that most *D. bruxellensis* strains are able to grow in environments with ethanol concentrations as high as 15% (*v*/*v*). Temperatures exceeding 36 °C over a period of 12 h resulted in complete loss of viability [82]. Additionally, these yeasts are able to survive and grow in environments with low pH and nutrients [69,78,81], although these environmental stresses can cause the yeasts to change into a viable but non-culturable (VBNC) state. While in this dormant state, the cells cannot be cultured without resuscitation even though they are still alive. Some researchers have found that this can sometimes occur directly after the addition of sulfur dioxide [69].

### 3.2. Other Spoilage Yeasts

*Zygosaccharomyces bailii* is another yeast that can cause cloudiness in bottled wine through the formation of flocculants and granular deposits. It can also produce acetic acid and metabolize malic acid resulting in off-odors and pH increase. Due to *Z*. *bailii*’s high resistance to yeast inhibitors including sulfur dioxide and tolerance to high ethanol (18%) environments, it can be difficult to control this yeast in wine [2,83]. Film-like growths can form on the surface of wines due to the presence of *Saccharomyces cerevisiae, Saccharomyces bayanus*, *Zygosaccharomyces fermentati* and species of *Candida, Pichia* and *Hansenula* [83].

### 3.3. Bacteria

The number of bacteria found on grapes varies depending on their condition, with healthy fruit typically having considerably less than damaged grapes. Spoilage caused by lactic acid bacteria typically occurs in warm environments with a pH higher than 3.5 and insufficient sulfur dioxide. *Lactobacillus brevis* and *Oenococcus* *oeni* cause the transformation of tartaric acid to lactic acid, which leads to a rise in pH, a dull red-brown color in red wines, an increase in carbon dioxide, cloudiness, and the formation of viscous deposits and mousy off-odors. *L. brevis* and *Lactobacillus buchneri* can also cause bitterness. Furthermore, *O. oeni* and *Pediococcus* can cause ropiness, characterized by the flotation of silky threads in spoiled wines [2,83]. Since the 19th century, acetic acid bacteria, including strains of *Gluconobactera* and *Acetobacter*, have been known to cause the oxidation of ethanol to undesirable acetic acid and the oxidation of polyols to ketones. Due to the ability of these acetic acid bacteria to survive anaerobic conditions, they are able to grow in barreled and bottled wines. Bacteria of the genus *Bacillus* cause the formation of sediment and earthy, musty off-odors [83]. Bacteria can be kept under control in wine by maintaining a low pH and temperature environment, minimizing the concentration of oxygen and adding sulfur dioxide [83].

### 3.4. Molds

Molds including *Aspergillus, Penicillium, Alternaria, Botrytis, Cladosporium, Mucor, Oidium, Plasmopara, Rhizopus* and *Uncinula* are known to infect grapes. These can enter in the process in the crushing stage, decreasing the juice yield and increasing the grape pressing time. Molds deteriorate the wine quality by altering its composition, producing off-flavors, and encouraging the undesirable growth of spoilage yeasts and bacteria. The resistance of molds to HPP is very variable, depending on the species [84]. Molds can easily be controlled in wine as they are unable to survive due to their susceptibility to alcohol concentration of ≥ 3% and SO_2_ [2].

## 4. Effect of PEF, HPP and Other Non-Thermal Technologies on Microbial Inactivation in Wine

This literature review showed limited studies of microbial inactivation in wine by some of the seven non-thermal technologies described previously in Section 2. Therefore, this section presents results of microbial inactivation in wine by PEF, HPP and US, and allows a comparison of the efficiency of each technology. The microbial inactivation for different non-thermal PEF, HPP and US conditions is expressed in terms of log reductions and shown in Table 1, Table 2 and Table 3. The wine composition, the microorganism species and strain are factors that affect the level of inactivation. For example, the wines’ electrical conductivity is directed related to its composition, and is crucial for PEF efficiency. The microbial reduction is expected to increase with process intensity and duration (time). Depending on the process, the intensity is given by the electric field strength for PEF (kV/cm), the pressure for HPP (MPa) and the acoustic power density for US (W/mL). With respect to continuous PEF processing, the real treatment time is calculated from the residence time (dependent on the flow rate), the frequency of the pulses, and the pulse width, with low flow rate conditions maximizing the treatment time. Being a batch process, the HPP conditions are straightforward, i.e., the pressure and time set for the constant pressure phase of the HPP cycle. The US can be set in batch or continuous mode.

### 4.1. Brettanomyces Bruxellensis

Table 1 shows a summary of *Brett* inactivation expressed in terms of log reductions for different non-thermal PEF, HPP and US conditions. An electric field strength of 20 kV/cm applied to red wine for 6000 μs, led to more than 4.8 log reductions of *Brettanomyces bruxellensis* [19]. Puértolas et al. (2009) [17] achieved 5.2 log reductions of *Dekkera bruxellensis* and 5.8 log reductions of *Dekkera anomala* in red wine using 100 pulses at 31 kV/cm. These results suggest that *D. bruxellensis* in more resistant to PEF inactivation than *D. anomala*. Van Wyk et al. (2019) [12] could reduce the treatment time to as low as 39 µs by increasing the electric field intensity to 50 kV/cm, to obtain 3.0 log reductions in *B. bruxellensis.*

Non-thermal HPP treatment at 400 MPa for only 5 s resulted in the complete inactivation (>7.0 log reductions) of *Brettanomyces bruxellensis* in Cabernet Sauvignon wine [11]. The same study concluded that the strain of *B. bruxellensis* had a significant effect on HPP inactivation. Strain AWRI 1499 proved to be the most resistant, with 3.0 log reductions in red wine after processing at 150 MPa for 10 min. Puig et al. (2003) [3] achieved at least 6.0 log reductions of *B. bruxellensis* using 500 MPa for 5 min (=300 s). Treatment at 100 MPa resulted in no significant *B. bruxellensis* inactivation [11,85]. This suggests a minimum threshold pressure below which no inactivation occurs. The results confirm the microbial inactivation dependence on HPP pressure and time [11,85,86]. Van Wyk & Silva (2017a) [10] investigated the effect of wine intrinsic properties on the inactivation of *B. bruxellensis*, by performing HPP studies in seven different wines, including red, white and rosé wines. HPP treatments at 200 MPa for 3 min resulted in 3.0, 3.8, 5.0, 5.8 and 6.0 log reductions in Dolcetto Syrah, SO_2_-free Cabernet Merlot, Syrah, Cabernet Sauvignon and Pinot Noir, respectively. Complete inactivation (>6.0 log reductions) was achieved in rosé wine using 200 MPa for 2 min, while only 15 s was required to achieve complete inactivation (>7.0 log reductions) in the Chardonnay wine [10], showing the effect of wine composition on *Brett* inactivation. Additionally, results showed that alcohol concentrations above 12.0% *v*/*v* had a significant effect on *Brett* inactivation with an increase of log reduction from 3.0 for 10.5–12% to 4.2 for 14% red Dolcetto Syrah wines, while wine pH from 3 to 4 in Cabernet Sauvignon wine was found to have no effect on *B. bruxellensis* inactivation [10].

Ultrasound (US) set at a low acoustic power density of 0.2 W/mL was not efficient for *Brett* inactivation, even after a long processing time of 20 min, which only reduced the yeast in 0.24 log in red wine [53]. When using thermo-sonication, the combination of thermal conditions of 50 °C with US treatment for 1 min, Gracin et al. (2016) [54] achieved 3.0 log reductions of *Brettanomyces bruxellensis* yeast in red wine and 2.0 log reductions of lactic acid bacteria. However, high temperature has a negative impact on wine sensory properties and is not recommended.

Research has shown that LEC is an effective technology for the inactivation of wine spoilage microorganisms. Lustrato et al. (2010) [73] investigated the inactivation of *D. bruxellensis* by LEC in red wine (13.5% alcohol) using 200 mA for 60 days. After 1 and 30 days, 4.0 and 5.2 log reductions were achieved, respectively [73]. Lustrato et al. (2015) [75] found that, after 30 days storage in oak barrels, there was no significant difference in the viable cell count of *D. bruxellensis* between the LEC treated (200 mA, <15 °C) wine and wine treated using SO_2_. At the end of the experiment, SEM (scanned electron microscope) images showed the rupturing of the yeast cells which caused its irreversible inactivation [75].

### 4.2. Yeasts Important for Wine

Table 2 shows a summary of inactivation of different yeasts in wine submitted to different technologies and processing conditions. Abca and Evrendilek (2014) [20] found that 31 kV/cm bipolar pulses resulted in 4.5 log reductions of *Saccharomyces cerevisiae* in red wine. The same electric field strength applied to *Saccharomyces bayanus* in red wine led to significantly higher inactivation of 5.4 log reductions [17]. Abca and Evrendilek (2014) [20] also looked at the inactivation of *Candida lipolytica* and *Hansenula anomala* in red wine and found that 31 kV/cm caused 4.4 and 3.2 log reductions, respectively. Thus *H. anomala* was more resistant to PEF than *C. lipolyitica* and *S. cerevisiae*.

Similar to the *B. bruxellensis* results, Puig et al. (2003) [9] achieved 6.0 log reduction of *Saccharomyces cerevisiae* in red and white wine using 500 MPa for 5 min. With a reduced pressure of 300 MPa for 6 min, Tonello et al. (1998) [86] achieved more than 7.0 log reductions of *S. cerevisiae* in wine. Tonello et al. (1996) [87] found that HPP treatment at 321 MPa for 180 s resulted in more than 7.0 log reductions. Using 400 MPa for 20 s, Tonello et al. (1996) [87] attained more than 3.5 log reductions of *S. cerevisiae* in white wine and >3.7 log reductions of *S. ludwigii* in rosé wine, respectively. It was also concluded that HPP inactivation depends on the type and size of microorganism targeted [87]. Environmental scanning electron microscopy analysis images of *S. cerevisiae* spores demonstrated its death after HPP treatment of 600 MPa for 5 min, showing the release of intracellular content and change in the shape and size of the cell [40]. Lastly, alcohol concentration proved to be an important factor for microbial inactivation, with higher log reductions achieved in wines with alcohol concentrations above 13% *v*/*v* [10,86,87].

Residual yeast inactivation (≤0.6 log reductions) was registered in red wine, even after a very long and unrealistic US treatment time of 20 min at 0.2 W/mL [53]. The highest yeast inactivation was a 0.6 log reduction of *Pichia membranefaciens* and the lowest a 0.13 log reduction of *Schizosaccharomyces pombe*.

HPH treatments of 150 MPa was able to induce a 2.2 log reduction of *Saccharomyces bayanus* [67]. Subsequently, further studies of Comuzzo et al. [88,89] revealed 3.5 log reductions using multiple HPH passes (10 L/h) at <40 °C. Puig et al. (2008) [68] found that subjecting red and white grape musts to 200 MPa using a flow rate of 120 L/h, caused complete inactivation of wild yeasts and lactic acid bacteria.

**Table 2:** Inactivation of different yeasts in wine by non-thermal PEF, HPP and US technologies *.

Yeast Species	Pasteurization Process	Wine	Alcohol Content (% *v*/*v*)	Processing Conditions	Treatment Time	Log Reduction	Reference
*Saccharomyces cerevisiae*	PEF	Red	12.0	31 kV/cm, 3 µs square bipolar pulse, 40 mL/min, T ≤ 40 °C	−	4.5	[20]
*Saccharomyces bayanus*	PEF	Red	13.0	31 kV/cm, 1 Hz, 100 pulses, batch, T < 30 °C	−	5.4	[17]
*Candida lipolytica*	PEF	Red	12.0	31 kV/cm, 3 µs square bipolar pulse, 40 mL/min, T ≤ 40 °C	−	4.4	[20]
*Hansenula anomala*	PEF	Red	12.0	31 kV/cm, 3 µs square bipolar pulse, 40 mL/min, T ≤ 40 °C	−	3.2	[20]
*Saccharomyces cerevisiae*	HPP	nr	15.0	300 MPa	360 s	>7.0	[86]
*Saccharomyces cerevisiae*	HPP	Red & white	nr	500 MPa	300 s	6.0	[9]
*Saccharomyces cerevisiae*	HPP	White	nr	400 MPa	20 s	>3.5	[87]
*Saccharomyces ludwigii*	HPP	Rosé	nr	400 MPa	20 s	>3.7	[87]
*Saccharomyces cerevisiae*	US	Red	14.0	24 kHz, 0.2 W/mL, T ≤ 25 °C	20 min	0.30	[53]
*Schizosaccharomyces pombe*	US	Red	14.0	24 kHz, 0.2 W/mL, T ≤ 25 °C	20 min	0.13	[53]
*Zygosaccharomyces bailii*	US	Red	14.0	24 kHz, 0.2 W/mL, T ≤ 25 °C	20 min	No inactivation	[53]
*Pichia membranefaciens*	US	Red	14.0	24 kHz, 0.2 W/mL, T ≤ 25 °C	20 min	0.60	[53]

* HPP was carried out at room temperature, maintaining nonthermal conditions; PEF—pulsed electric fields; HPP—high pressure processing; US—power ultrasound, nr—not reported.

### 4.3. Bacteria

Table 3 shows the results of bacteria inactivation in wine by non-thermal technologies. Only 2.7 log reductions of *Lactobacillus delbrueckii* ssp. *bulgaricus* was achieved in red wine using 31 kV/cm bipolar pulses [20]. Puértolas et al. (2009) [17] treated red wine containing *Lactobacillus plantarum* and *Lactobacillus hilgardii* using 100 pulses at 31 kV/cm, resulting in 4.8 and 5.2 log reductions, respectively. The magnitude of bacteria inactivation was similar or slightly lower than with yeasts (5.2 to 5.8 log reductions), using the same process. Lastly, 20 kV/cm applied for 6000 μs led to >1.0 and >5.3 log reductions of *Pediococcus parvulus* and *Oenococcus oeni* in red wine, respectively [19]. Therefore, research has shown that the size of the microorganisms has a significant effect on PEF inactivation, with larger yeast cells being less resistant to inactivation than smaller bacteria cells [17,20].

**Table 3:** Inactivation of bacteria in wine by non-thermal PEF, HPP and US technologies *.

Bacterium Species	Pasteurization Process	Wine	Alcohol Content (% *v*/*v*)	Processing Conditions	Treatment Time	Log Reduction	Reference
*Lactobacillus* *plantarum*	PEF	Red	13.0	31 kV/cm, 1 Hz, 100 pulses, batch, T < 30 °C	−	4.8	[17]
*Lactobacillus hilgardii*	PEF	Red	13.0	31 kV/cm, 1 Hz, 100 pulses, batch, T < 30 °C	−	5.2	[17]
*Lactobacillus* *delbrueckii* *ssp. bulgaricus*	PEF	Red	12.0	31 kV/cm, 3 µs square bipolar pulse, 40 mL/min, T ≤ 40 °C	−	2.7	[20]
*Pediococcus parvulus*	PEF	Red	nr	20 kV/cm, 0.5 Hz, 10 µs pulse width, T ≤ 42 °C	6000 µs	>1.0	[19]
*Oenococcus oeni*	PEF	nr	nr	20 kV/cm, 0.5 Hz, 10 µs pulse width, T ≤ 38 °C	6000 µs	>5.3	[19]
*Lactobacillus* *plantarum*	HPP	Red & white	nr	500 MPa	300 s	8.0	[9]
*Pediococcus* *damnosus*	HPP	Red	nr	400 MPa	20 s	>3.4	[87]
*Oenococcus oeni*	HPP	Red & white	nr	500 MPa	300 s	8.0	[9]
*Acetobacter aceti*	HPP	Red & white	nr	500 MPa	300 s	8.0	[9]
*Acetobacter aceti*	HPP	Red	nr	400 MPa	20 s	>4.2	[87]
*Acetobacter* *pasteurianus*	HPP	Red & white	nr	500 MPa	300 s	8.0	[9]
*Lactobacillus* *plantarum*	US	Red	14.0	24 kHz, 0.2 W/mL, T ≤ 25 °C	20 min	0.13	[53]
*Pediococcus* sp.	US	Red	14.0	24 kHz, 0.2 W/mL, T ≤ 25 °C	20 min	0.35	[53]
*Oenococcus oeni*	US	Red	14.0	24 kHz, 0.2 W/mL, T ≤ 25 °C	20 min	0.22	[53]
*Acetobacter* *pasteurianus*	US	Red	14.0	24 kHz, 0.2 W/mL, T ≤ 25 °C	20 min	0.60	[53]

* HPP was carried out at room temperature, maintaining nonthermal conditions; PEF—pulsed electric fields; HPP—high pressure processing; US—power ultrasound, nr—not reported.

A number of HPP inactivation studies have been conducted on spoilage bacteria in red and white wines (Table 3). Puig et al. (2003) [9] used 500 MPa for 5 min to investigate the inactivation of *Lactobacillus plantarum, Acetobacter aceti*, *Acetobacter pasteurianus* and *Oenococcus oeni* in red and white wine, resulting in 8.0 log reductions for all four bacteria. Tonello et al. (1996) [87] found that 400 MPa applied for only 20 s resulted in >4.2 log reductions of *A. aceti* in wine. Tonello et al. (1996) [87] also looked at the inactivation of *Pediococcus damnosus* in wine using 400 MPa for 20 s, which led to more than 3.4 log reductions.

Regarding US, similarly to yeast, the inactivation of bacteria was almost none, ranging from log reductions of 0.13 with *Lactobacillus plantarum* to 0.35 with *Pediococcus* sp. bacteria [53] after a 20 min treatment (0.2 W/mL). 

## 5. Comparison of Technologies and Final Remarks

PEF and HPP proved to be effective wine pasteurization technologies, as they inactivate key wine spoilage yeasts and bacteria in short periods of time, feasible for application in the wine industry. Both technologies have the potential to complement or be used as alternatives to SO_2_ addition to must, grape juice and finished wine at different stages of wine production, to control undesirable microbial growth or stop fermentation, and stabilize and preserve the quality of the finished wine until consumption. In fact, PEF is a promising technology for the wine industry as it is a continuous technology, requiring short processing times, in the magnitude of microseconds, for the inactivation of microbes of concern in the wineries. This enables commercial scale production with higher throughput. In addition, the same PEF unit also has the potential to decrease wine maceration time during the early stages of production. HPP and US have been investigated for the acceleration of wine ageing, reducing the required vinification time. US produced insufficient inactivation even after application of unrealistically long processing times.

Despite the encouraging results demonstrating less or no SO_2_ addition to wine by using non-thermal technologies such as HPP [90] and PEF [91], more research is needed to determine the extent to which the use of SO_2_ can be reduced or eliminated in the production/stabilization of different types of wine. The role of SO_2_ in wine is complex and more research is required involving simultaneous assessment of microbial inactivation and wine quality after processing and during storage. Further wine stability studies with SO_2_ free wines are needed to compare the quality of the wine produced using non-thermal methods vs the conventional addition of SO_2_. More wine stability/quality studies should focus on the combination of a non-thermal method with a reduced amount of added SO_2_ preservative.

Another important aspect is the investigation and comparison of costs of non-thermal technologies in terms of capital investment, energy requirement and environmental impact. In addition, although some technologies such as HPP are already used at commercial scale for other beverages and packed foods, others are not. Fortunately, the modern wine consumer’s increasing demand for healthier and preservative free novel wines serves to promote research as well as applications and improvements of non-thermal technologies to the wine industry.

This review shows the potential of both HPP and PEF for wine preservation, as these technologies have minimal effect on overall wine sensory quality (flavor and aroma) and biochemical quality factors such as antioxidant activity, phenolic content and anthocyanins.

## Figures and Tables

**Table 1:** Inactivation of *Brettanomyces bruxellensis* yeast in wine by non-thermal PEF, HPP and US technologies *.

Pasteurization Technology	Wine	Alcohol Content (% *v*/*v*)	Processing Conditions	Treatment Time	Log Reduction	Reference
PEF	Red	13.0	31 kV/cm, 1 Hz, 100 pulses, batch, T < 30 °C	−	5.2	[17]
PEF	Red	nr	20 kV/cm, 0.5 Hz, 10 µs pulse width, T ≤ 37 °C	6000 µs	>4.8	[19]
PEF	Red	13.5	50 kV/cm, 100 Hz, 1.7 µs pulse width, T < 40 °C	39 µs	3.0	[12]
HPP	Red Cabernet Sauvignon	13.4	400 MPa	5 s	>7.0	[11]
HPP	White Chardonnay	13.0	200 MPa	15 s	>7.0	[10]
HPP	Rosé	12.5	200 MPa	120 s	>6.0	[10]
HPP	Red Pinot Noir	13.0	200 MPa	180 s	6.0	[10]
HPP	Red & white	nr	500 MPa	300 s	6.0	[9]
HPP	Red Cabernet Sauvignon	13.5	200 MPa	180 s	5.8	[10]
HPP	Red Syrah	12.5	200 MPa	180 s	5.0	[10]
HPP	Red SO_2_-free Cabernet Merlot	13.7	200 MPa	180 s	3.8	[10]
HPP	Red Dolcetto Syrah	10.5 14.0	200 MPa	180 s	3.0 4.2	[10]
US	Red	14.0	24 kHz, 0.2 W/mL, T ≤ 25 °C	20 min	0.24	[53]

* HPP was carried out at room temperature, maintaining nonthermal conditions; PEF—pulsed electric fields; HPP—high pressure processing; US—power ultrasound, nr—not reported.
